# Descriptive epidemiological study on patients with movement disorders, with emphasis on Parkinson’s disease

**DOI:** 10.1590/1516-3180.2020.0119.R1.30102020

**Published:** 2020-12-23

**Authors:** Luiza Serafini Balestrassi, Sonia Maria Cesar Azevedo Silva

**Affiliations:** I MD, MSc. Neurologist, Department of Neurology, Hospital do Servidor Público Estadual (IAMSPE), São Paulo (SP), Brazil.; II MD, PhD. Neurologist, Movement Disorders Unit, Department of Neurology, Universidade Federal de São Paulo (UNIFESP), São Paulo (SP), Brazil.

**Keywords:** Movement disorders, Epidemiology, Parkinson’s disease, Hypokinesia, Extrapyramidal symptoms, Parkinsonism, Hyperkinetic syndromes

## Abstract

**BACKGROUND:**

Knowing the epidemiological profile is relevant for improving healthcare practices. Movement disorders are neurological disorders characterized by the presence of involuntary movements. They have a negative impact on patients’ quality of life.

**OBJECTIVES:**

To outline the frequencies of the different diagnoses seen among patients, along with their demographic characteristics, at a hospital in São Paulo (SP), Brazil, and to highlight the clinical aspects of those with Parkinson’s disease.

**DESIGN AND SETTING:**

Retrospective descriptive epidemiological analysis at a specialized outpatient clinic in a state public hospital in São Paulo.

**METHODS:**

Patients treated at this clinic over a four-year period were analyzed. Diagnoses, demographic variables and associations with clinical aspects of Parkinson’s disease were evaluated.

**RESULTS:**

Out of the 680 medical records analyzed, 58.4% related to females. Most patients were over 60 years of age, white, married and teachers. The most frequent diagnosis was Parkinson’s disease, followed by essential tremor and dystonia. Parkinson’s disease presented in the mixed clinical form; the most common initial symptom was tremor. The akinetic-rigid clinical form occurred in younger individuals and mostly presented with postural instability and freezing of gait in the early years of disease.

**CONCLUSIONS:**

Parkinson’s disease, essential tremor and dystonia were the most frequent diagnoses. Characteristics like sex, frequency of other pathological conditions and the clinical and demographic aspects of Parkinson’s disease were consistent with the data in the relevant literature.

## INTRODUCTION

Epidemiological studies are widely reported in the literature,^1^^,^^2^^,^^3^^,^^4^ and are extremely valuable for improving healthcare practices. Epidemiological information is an instrument for healthcare planning that informs guidelines and improvements.^5^^,^^6^^,^^7^ Consequently, analysis on certain factors such as risk, morbidity and mortality, prevalence of certain diseases and assistance provided to patients is required.^5^ Such information facilitates identification of a population profile and allows comparison of characteristics with data in other studies that have been consolidated in the literature.^6^^,^^7^^,^^8^

Some studies on neurological diseases have revealed that one of the most recurrent diagnoses consists of movement disorders.^9^ These are neurological disorders resulting from lesions in the basal nuclei, which result in involuntary movements.^10^ These disorders are broadly classified into two major groups: hyperkinetic and hypokinetic syndromes. Hyperkinetic syndromes are characterized by hyperactive involuntary and uncontrollable movements, such as ballism, athetosis, dystonia, tremor, tics, myoclonus and others.^11^^,^^12^^,^^13^ Antagonistically, hypokinetic syndromes, for which the prototype is Parkinson’s disease, are characterized by slowness and decreased voluntary movements (bradykinesia/akinesia), in addition to muscle stiffness, resting tremor and postural instability.^11^^,^^12^^,^^13^ This group also includes other parkinsonian syndromes, such as progressive supranuclear palsy (PSP), multiple system atrophy (MSA), corticobasal degeneration and vascular and drug-induced secondary parkinsonism.^14^

Parkinson’s disease is considered to be the second most common degenerative disorder after Alzheimer’s disease.^15^ Although its etiology is not yet well defined, it is characterized by degeneration of dopaminergic neurons in the nigrostriatal system of the basal nuclei.^16^ The prevalence of Parkinson’s disease in the general population has been estimated to be 0.3%,^17^ such that it affects approximately 1% of individuals over 60 years of age^15^^,^^16^^,^^17^ and 4% to 5% of those over 85 years of age.^15^

Progressive supranuclear palsy is considered to be the second most common form within the range of parkinsonian syndromes. Its incidence is approximately 5.3 cases per 100,000 people between 50 and 99 years old, and its prevalence ranges from 4.9 to 6.5/100,000.^18^ Another example of parkinsonian syndrome is MSA, with an estimated incidence of 0.6 cases per 100,000 people, and a prevalence of 1.86-4/100,000, although there are few published studies on this.^15^^,^^19^ Additionally, several other movement disorder-related diagnoses exist, thus highlighting the importance of evaluating their behavior.

These data are necessary for evaluating neurological institutions and services, and are important for identifying risk factors and geographical differences that might influence the development of treatment strategies. Knowledge of the characteristics and prevalence of diagnoses in this environment is required, in order to make comparisons with studies published previously.

## OBJECTIVE

The objective of this study was to outline the frequencies of the different diagnoses seen among patients, along with their demographic characteristics, at the movement disorders outpatient clinic of a state public hospital, in São Paulo (SP), Brazil, and to highlight the clinical aspects of those with Parkinson’s disease.

## METHODS

This study consisted of a retrospective descriptive epidemiological review of data obtained from the medical records of patients seen at the neurology outpatient clinic specializing in movement disorders in a state public hospital, in São Paulo, Brazil. The medical records were searched based on a survey of patients with scheduled appointments who were seen at the movement disorders outpatient clinic. The analysis period comprised four years, starting from December 2009, when an electronic appointment scheduling system was implemented. The data were collected after the study protocol had been approved by an Internal Review Board (Ethics Committee) on May 13, 2014 (number 668.444/ CAAE: 305294 14.6.0000.5463) and had been authorized by the person in charge of the Medical Archive and Statistics Service (SAME).

Analysis on the medical records was previously authorized and scheduled as a maximum number of 20 medical records per day. The records were analyzed individually in the medical file sector of the state public hospital, by one of the doctors responsible for this research. The study exclusively included patients who had been diagnosed with movement disorders.

The patients’ diagnoses were recorded from the medical records. The clinical evolution of the anamnesis and neurological examination were analyzed based on the diagnostic criteria. This information was described by neurology specialists in the movement disorder department. The entire procedure was supervised by chief neurologists.

The following demographic data were collected: age, sex, skin color, profession and marital status. In addition, correlations of the following relationships were assessed: “clinical diagnosis versus age”; and “clinical diagnosis versus sex”.

Considering that the most frequent etiology would be Parkinson’s disease, the following clinical characteristics were evaluated: initial symptoms, clinical form of the disease, freezing of gait and postural instability within the first five years of the disease. In addition, the following clinical correlations were considered: “clinical form versus age”; and “clinical form versus freezing of gait and postural instability”.

The retrospective nature of this study was characterized as a limitation, considering that there was therefore a possibility of missing information. It is important to highlight that when information was not explicitly reported, these data were classified as not described (ND). Patients who did not have a defined etiological diagnosis due to lack of follow-up at the clinic or insufficient information were classified as “unspecified”.

The data were expressed as absolute and relative frequencies for adequate statistical treatments. The chi-square and Fisher’s exact tests were applied, depending on the distribution of values in the groups. The Stata statistical software, version 13.1, (StataCorp, 2013: Stata Statistical Software, Release 13; College Station, TX, United States) was used in all analyses and the confidence level was set at 95%, i.e. a P-value < 0.05 was considered significant. It is important to note that, when the total number of cases evaluated that were described as “ND” was less than 10%, these were not considered in the study because they were not statistically significant.

## RESULTS

Out of the 790 medical records analyzed, 680 were selected because they involved a diagnosis of movement disorders. Of these, 277 records included a diagnosis of Parkinson’s disease. Thus, in order to demonstrate the analysis more clearly, the results are presented in two stages: (1) Parkinson’s disease analysis; and (2) diagnoses of other movement disorders.

### Parkinson’s disease

We observed higher occurrence of the mixed clinical form of Parkinson’s disease, which was present in 77.6% of all of these patients. The tremulous (TREM) and akinetic-rigid (AR) forms accounted for 18.4% of the case, and the other 4% of the medical records of Parkinson’s disease did not specify the form. The most common initial symptom reported by the patient was tremor. It is important to emphasize that the diagnoses of postural instability and freezing of gait were not present in the first five years of follow-up, as described in Table 1.

**Table 1.** Distribution of patients with Parkinson’s disease treated in the movement disorders outpatient clinic of a state public hospital, São Paulo (SP), 2017

Total	n	%
	277	100
Clinical form of the disease		
Akinetic-rigid	36	13
Tremulous	15	5.4
Mixed	215	77.6
Not described	11	4
Initial symptom of the disease		
Tremor	167	60.3
Rigidity	56	20.2
Postural instability	9	3.3
Bradykinesia	7	2.5
Not described	38	13.7
Postural instability (first 5 years of the disease)		
No	148	53.4
Yes	88	31.8
Not described	41	14.8
Freezing of gait (first 5 years of the disease)		
No	164	59.2
Yes	30	10.8
Not described	83	30

Source: research data.

The Parkinson’s disease analysis showed that 66.7% of the patients above 61 years of age were diagnosed with the mixed form, and 71.4% of the patients below 61 years were diagnosed with the AR form. In 46.1 of the patients with the TREM form, the onset was when they were above 71 years old.

Table 2 shows that there was an association between early diagnosis and the AR form. We found that the older the patient was at diagnosis, the greater the probability of occurrence of the mixed and TREM forms was.

**Table 2.** Analysis of the association between the clinical form of Parkinson’s disease and age at diagnosis, postural instability and freezing of gait among patients diagnosed with Parkinson’s disease who were treated at the movement disorders outpatient clinic of a state public hospital, São Paulo (SP), 2017

	Form of Parkinson’s disease			P-value
	AR	TREM	Mixed	
	n (%)	n (%)	n (%)	
Age at diagnosis				
< 61 years	25 (71.4)	2 (15.4)	70 (33.3)	< 0.001
61-70 years	4 (11.4)	5 (38.5)	76 (36.2)	
71 years or older	6 (17.2)	6 (46.1)	64 (30.5)	
Postural instability				
No	16 (48.5)	12 (100)	118 (62.8)	0.007
Yes	17 (51.5)	0 (0)	70 (37.2)	
Freezing of gait				
No	16 (69.6)	11 (100)	135 (86.0)	0.043
Yes	7 (30.4)	0 (0)	22 (14.0)	

Source: research data. AR = akinetic-rigid; TREM = tremulous.

No individual with the TREM form in our sample presented postural instability and freezing of gait within the first five years of the disease. This characteristic was also observed in most patients with the mixed form. Furthermore, there was higher relative frequency of instability and freezing of gait among individuals with the AR form, thus indicating less favorable conditions for those patients.

The results from this analysis were compared with studies in the literature. This demonstrated that similar methodologies had been used to study Parkinson’s disease in different parts of the world. The frequencies of occurrence described in previously published reports^5^^,^^6^^,^^11^^,^^14^ suggested that Parkinson’s disease was the most common cause of movement disorders in the entire population studied, which was consistent with the results from our analysis. However, an increasing number of secondary and atypical diagnoses of parkinsonism exist and need to be considered. Consequently, an analysis of other movement disorder diagnoses is presented in the following section, in detail.

### Analysis on other movement disorder diagnoses

The results from the analysis on the data collected during the study period showed that the most frequent diagnoses were Parkinson’s disease (40.7%), essential tremor (15.4%) and dystonia (13.1%). The remaining diagnoses accounted for 33.6% of the cases. The frequencies of syndromic and etiological diagnoses of movement disorders are described in Table 3. The pattern shown in Table 3 was also observed in several similar studies conducted in other regions,^5^^,^^6^^,^^10^^,^^11^^,^^12^^,^^13^ including the study by Bhidayasiri et al.^7^ and a study at the Columbia University Medical Center in conjunction with the Baylor College of Medicine, in which approximately 43,000 patients were analyzed.^14^

**Table 3.** Frequencies of diagnoses among patients treated in the movement disorders outpatient clinic of a state public hospital, São Paulo (SP), 2017

Total	N	Relative %	Total %
	680	100	100
Parkinsonism (n = 336)			
Parkinson’s disease	277	81.7	40.7
Atypical parkinsonism	12	3.5	1.8
Secondary parkinsonism	19	5.6	2.8
Not specified	28	9.1	4.1
Tremor (n = 141)			
Essential	97	68.8	14.3
Drug-induced	4	2.8	0.6
Not specified	26	18.4	3.8
Others	14	10.0	2.1
Dystonia (n = 89)			
Primary	89	100	13.1
Myoclonus (n = 61)			
Hemifacial spasm	61	100	9.0
Ataxia (n = 8)			
Spinocerebellar ataxia type 2	1	12.5	0.1
Spinocerebellar ataxia type 3	1	12.5	0.1
Spinocerebellar ataxia type 6	2	25	0.3
Not specified	4	50	0.6
Chorea (n = 17)			
Huntington’s disease	7	41.2	1.0
Huntington-like 2	1	5.9	0.1
Vascular	5	29.4	0.7
Not specified	4	23.3	0.6
Tardive dyskinesia (n = 5)			
Tardive dyskinesia	5	100	0.7
Restless leg syndrome (n = 13)			
Restless leg syndrome	13	100	1.9
Tics (n = 10)			
Tourette’s disease	2	20	0.3
Others	8	80	1.2

Source: research data.

In our study, a diagnosis of parkinsonism was described in 336 records, of which 81.7% were attributed to Parkinson’s disease. The other diagnoses included 9.1% with no defined etiology, which was classified as unspecified parkinsonism. Out of that total, 5.6% were diagnosed with drug-induced (n = 11) and vascular (n = 8) secondary parkinsonism. Atypical parkinsonism had an incidence of 3.5%, consisting of Lewy body disease (LBD) (n = 4), multiple system atrophy (n = 4) and progressive supranuclear palsy (n = 4).

The second most frequent pathological condition documented in the present study was 97 cases of essential tremor, which is one of the most common diagnoses in neurology outpatient clinics, often surpassing the number of Parkinson’s disease cases. Its worldwide prevalence is widely variable, with descriptions ranging from one to more than 200 cases per 1,000 individuals.^15^ Other etiologies for tremor were found in 31.2% of the cases. These cases included the following types: unspecified (n = 26), drug-induced (n = 4), others represented by a specific writing task (n = 3), Holmes (n = 2), orthostatic (n = 1) and psychogenic (n = 8).

Dystonia, which had the highest incidence, is a frequent condition reported in most studies,^5^^,^^8^ and was diagnosed in 89 cases in our study, all corresponding to primary dystonia. Table 4 shows the classification of primary dystonia according to anatomical location. Out of the total number of cases analyzed, 78 were focal, four were generalized and six were segmental dystonia. The focal dystonia cases accounted for 37.2% and corresponded to the cervical type, followed by 23.1% with writer’s cramps, 17.9% each for upper-limb and blepharospasm cases and 3.8% for oromandibular cases.

**Table 4.** Demographic characteristics of patients treated at the movement disorders outpatient clinic of a state public hospital, São Paulo (SP), 2017

Total	680 (n)	100 (%)
Sex		
Female	397	58.4
Male	283	41.6
Age		
< 30 years	2	0.3
31 to 50 years	51	7.5
51 to 60 years	93	13.7
61 to 70 years	175	25.7
71 to 80 years	213	31.3
81 years or more	146	21.5
Race		
White	576	84.8
Mulatto	23	3.4
Black	45	6.6
Not described	36	5.3
Marital status		
Married	418	64.4
Divorced	42	6.2
Single	108	15.9
Widower	81	11.9
Not described	31	4.6
Profession		
Teacher	110	16.2
Housewife/husband	80	11.8
Service assistant	15	2.2
Others	231	33.9
Not described	244	35.9

Source: research data.

The presence of dystonia has also been reported in the literature, at rates ranging from 2-50 cases per million (early onset dystonia) to 30-7,320 cases per million (late onset dystonia). This reflects the need for new epidemiological studies such as the present one.^12^^,^^13^ According to the published reports, the occurrence rate has not changed over the years. Siemers and Reddy^11^ reported dystonia in 17% of their movement disorder cases, which was a higher prevalence than that of their tremor cases (12%).

In the present study, chorea was present in 2.4% of the cases, Huntington’s disease accounted for 1% of the cases and other forms such as vascular, Huntington-like type 2 and unspecified chorea totaled 1.4%. Ataxia had an occurrence of 1.1%, and the diagnoses of restless leg syndrome, tardive dyskinesia and tics represented 4.1% in our sample.

The demographic data, described in Table 4, showed that 58.4% of the patients were female and 41.6% were male. There was greatest frequency (31.3%) of the 71 to 80-year age group, followed by 61 to 70 years (25.7%) and above 80 years (21.5%). We found that 84.8% of the patient sample self-identified as white, and 64.4% were married. The most frequent professional activity was as a teacher (16.2%), followed by housewife/husband (11.8%) and general service assistant (2.2%). However, 35.9% of the medical records did not have any information on professional activity.

Both sexes exhibited high frequency of parkinsonism, followed by tremor. However, proportionally higher frequencies of restless leg syndrome (76.9%), dystonia (73%) and myoclonus (65.6%) were present in female patients, while tic (60%) and tardive dyskinesia (60 %) were more common in males. Parkinsonism had similar distribution between the male and female groups, as described in Table 5, which shows absolute and relative values.

**Table 5.** Distribution of movement disorder patients according to sex and age, São Paulo (SP), 2017

Diagnosis	PK	Tremor	RLS	Ataxia	Myoclonus	Chorea	TD	Dystonia	Tics
Sex									
Female	175 (52.1)	86 (61.0)	10 (76.9)	5 (62.5)	40 (65.6)	10 (58.8)	2 (40.0)	65 (73.0)	4 (40.0)
Male	161 (47.9)	55 (39.0)	3 (23.1)	3 (37.5)	21 (34.4)	7 (41.2)	3 (60.0)	24 (27.0)	6 (60.0)
Age (years)									
< 51	10 (3.0)	9 (6.8)	0 (0)	4 (50.0)	7 (11.5)	3 (17.6)	1 (20.0)	15 (16.9)	4 (40.0)
51-60	22 (6.5)	17 (12.9)	1 (7.7)	2 (25.0)	13 (21.3)	3 (17.6)	1 (20.0)	32 (36.0)	2 (20)
61-70	80 (23.8)	40 (30.3)	2 (15.4)	0 (0)	18 (29.5)	5 (29.4)	1 (20.0)	26 (29.2)	3 (30.0)
71-80	122 (36.3)	40 (30.3)	7 (53.8)	2 (25.0)	19 (31.1)	3 (17.6)	0 (0)	10 (11.2)	1 (10.0)
> 80	102 (30.4)	26 (19.7)	3 (23.1)	0 (0)	4 (6.6)	3 (17.6)	2 (40.0)	6 (6.7)	0 (0)

Source: research data. PK = parkinsonism; RLS: restless legs syndrome; TD = tardive dyskinesia.

The distribution of the diagnoses according to age group revealed a statistical difference between the groups listed. Parkinsonism was more frequent in the age groups above 60 years, while dystonia showed higher frequency in individuals aged 60 years or less. The other syndromes had uniform distribution, or were highly influenced by low sample sizes in the groups, e.g. the distribution of ataxia. These analyses are described in Table 5.

## DISCUSSION

Neuroepidemiology aims to determine the presence of neurological diseases in the population and acts as an instrument for healthcare planning and improvement. This has undergone increasing changes due to increases in life expectancy.

The retrospective use of medical records for data collection (included missing or incomplete data) was a limitation of this study. This can be explained in terms of difficulty in interpreting the documented information and variability in the quality of documentation among professionals. However, the most important information for the purpose of this study was indeed documented, i.e. the final diagnosis and clinical details.

This study demonstrated that high frequency of Parkinson’s disease was present. A previous study conducted in Brazil indicated that the incidence of this pathological condition was 68.9% among a total of 338 patients,^20^ a result that was similar to ours. Other studies have indicated high incidence of this diagnosis, in several countries, such as Thailand,^7^ Spain,^10^^,^^18^ United States,^11^ Mexico,^16^ India,^8^ Denmark^21^ and Brazil.^20^^,^^22^^,^^23^

In contrast, other research showed lower prevalences in Africa,^24^ China^25^ and some countries in Latin America, such as Argentina^26^ and Bolivia,^27^ compared with developed countries, especially in Europe. The demographic factors in this study showed that there was slight predominance among females, with an F:M ratio of approximately 1.03. On the contrary, other published reports have shown higher prevalence among males.^22^^,^^26^

Parkinson’s disease may be classified into three clinical forms: tremulous, akinetic-rigid and mixed. As indicated in this study, the mixed form is considered to be most frequent. This conclusion is corroborated by results found in other published studies.^14^^,^^28^^,^^29^ In our study, the clinical forms were correlated with age, and we showed that most patients presenting the tremulous form (84.6%) were over 60 years of age, thus confirming the results of Pagano et al.^30^ Approximately 71.4% of the patients with the akinetic-rigid form were diagnosed under the age of 60 years. Previous reports have demonstrated biological differences between the clinical forms of Parkinson’s disease.^14^^,^^31^

Resting tremor was reported in this study, in 60.3% of the patients at the onset of the disease. It is considered to be the most frequent initial symptom in the mixed and tremulous forms.^29^ Delval et al.^32^ showed that 27% of the patients analyzed in their study presented with a history of freezing of gait at the first evaluation, with a considerable increase observed eight years after the initial diagnosis.^33^ Other published reports confirmed the results from this study, thus suggesting that freezing of gait occurs with higher frequency in the akinetic-rigid form of the disease.^32^

As previously stated, the most frequent diagnosis in our study was Parkinson’s disease. However, increasing numbers of cases of secondary and atypical parkinsonism cases are common. This result was found in a previously published study,^7^ which showed that vascular parkinsonism was the second most frequent cause (4.9%), and that multiple system atrophy (MSA) and Lewy body disease (LBD) were the most common atypical parkinsonian syndromes (drug-induced parkinsonism was not found). However, studies in other countries have shown different patterns, depending on the region studied. Rodríguez-Violante et al.^16^ evaluated 647 patients in Mexico, among whom 46 were diagnosed with parkinsonism (atypical and secondary), representing 7.1% of a sample in which the most frequent diagnoses were vascular parkinsonism, progressive supranuclear palsy (PSP) and Lewy body disease.

Colosimo et al.^17^ conducted a longitudinal retrospective analysis on data from the PRIAMO study in Italy, in which 1,307 patients were diagnosed as presenting atypical and secondary parkinsonism. Among these, 6.4% had vascular parkinsonism, 2.6% MSA, 2.3% PSP, 1.1% LBD and 0.8% corticobasal degeneration (a diagnosis not present in the present study). Nevertheless, in a similar study conducted in Spain to evaluate the older population, it was reported that the most frequent etiologies were drug-induced and vascular parkinsonism.^18^

Those etiologies were also present in other recent studies. Savica et al.^19^ reported that out of 906 cases of parkinsonism in the United States, 11.9% corresponded to the drug-induced form, a result that differed from the present study. However, a review of previously published surveys conducted in Brazil showed predominance of secondary drug-induced and vascular parkinsonism.^20^^,^^22^ Notably, some of the studies mentioned above have presented considerable numbers of parkinsonism cases of undetermined etiology, thus emphasizing the need for standardization of clinical evaluations and for possible use of supplementary examinations for better etiological definition.

Hemifacial spasm is characterized by segmental facial myoclonus, which was the fourth most common diagnosis found in the present study. A previously published report by Batla et al.^34^ showed that the incidence of this condition was 7% in India. Other surveys conducted in the cities of Barcelona and Madrid indicated that the incidence of hemifacial spasm was 1.8% and 5%, respectively.^5^^,^^10^ Although hemifacial spasm is a common complaint in neurology outpatient clinics, its frequency does not differ significantly from that of other extrapyramidal diseases.^5^ This pattern was also observed in a study by Fahn et al.^14^ in the United States, which showed the same frequency of this pathological condition as found in the present study.

Our results for ataxia and chorea were similar to those in other published reports.^6^^,^^7^^,^^10^^,^^11^ However, one study in Ethiopia reported that the prevalence of ataxia was more than twice that of chorea, even surpassing the rate of diagnosing dystonia.^6^ These results suggest that there was a lack of uniformity in the data for making this diagnosis.

Huntington’s disease has been described as the primary cause of genetic chorea, which is the most prevalent type of chorea. A study conducted in the city of São Paulo, Brazil, on 119 medical records from patients diagnosed with chorea showed that 79% of the samples corresponded to Sydenham’s chorea, Huntington’s disease and vascular chorea.^35^ Another study conducted in Brazil on the genetic causes of chorea showed that the incidence of Huntington’s disease was 89.4%, of which 3.8% were Huntington-like type II cases, which is the second most common cause of chorea.^36^ Vascular chorea was the second most common cause of non-genetic chorea in the present study, thus confirming the results of Piccolo et al.,^37^ who reported that vascular chorea accounted for 41% of their cases in Italy.

The etiological classification of ataxias showed subtle predominance of spinocerebellar ataxia type 6 (SCA6), which has low prevalence in Brazil. This distribution differed from what has been reported in other countries, i.e. 30% prevalence in Australia, 28% in Japan and 15% in the United States.^38^ In our study, SCA2 and SCA3 both had incidences of 12.5%. Despite the low frequency in our study, these etiologies are recognized as the most frequent types.^39^ Similar results were also found in other studies.^38^^,^^39^^,^^40^

The diagnoses of tics and tardive dyskinesia were infrequent in our study, and also infrequent in other movement disorder outpatient clinics.^5^^,^^6^^,^^11^ Fahn et al.^14^ reported that tics and Tourette’s syndrome together accounted for the diagnoses of 6.4% out of a total of 2,753 patients in the United States. Similar results were also found in another study conducted in Brazil.^41^

Lastly, the demographic characteristics reported in the present study corroborated what was observed in most studies in the literature. Females predominated among the diagnoses of ataxia,^39^ chorea,^35^^,^^37^ myoclonus, parkinsonism,^23^ dystonia^12^ and tremor,^5^^,^^15^ while males predominated in the other cases. Movement disorders predominated among adult and older patients. The younger diagnostic groups were represented by ataxia, tics and dystonia.^5^^,^^6^^,^^8^

## CONCLUSIONS

Knowledge of the characteristics of movement disorder patients helps to model and promote better diagnostic and treatment strategies for this population. This study showed higher frequencies of diagnoses of Parkinson’s disease, essential tremor and dystonia, and the patients were predominantly white, female, aged between 71 and 80 years, teachers and married. The mixed clinical form of Parkinson’s disease with an initial symptom of resting tremor was found in higher numbers of patients. The akinetic-rigid (AR) clinical form of Parkinson’s disease tended to occur in younger individuals, with symptoms of postural instability and freezing of gait that developed earlier. On the other hand, the mixed and tremulous forms of Parkinson’s disease had greater likelihood of late onset. The present study showed that there is a need for more complete medical records, to enable better healthcare practices and epidemiological follow-up for patients. This would result in better diagnoses and strategies for analyzing the profile of patients with movement disorders.
